# Supplementary material for: Subsampled open-reference clustering creates consistent, comprehensive OTU definitions and scales to billions of sequences
Source: PeerJ. 2014 Aug 21;2:e545. doi: 10.7717/peerj.545 (PMC4145071; doi:10.7717/peerj.545)
Supplement: Supplemental Information 1 — Description of where to obtain data for this study. [file peerj-02-545-s001.docx]

## Data availability

The raw sequence data analyzed in this study is available in the QIIME Database under accession numbers 103 (88-soils), 449 (whole-body), and 550 (moving-pictures). All analyses were run with QIIME 1.8.0-dev. All commands, as well as all processed data and IPython Notebooks that illustrate how to work with that data are available in this project’s GitHub repository at <https://github.com/gregcaporaso/cloaked-octo-ninja>.
